# Factors that influence Cape fur seal predation on Cape gannets at Lambert’s Bay, South Africa

**DOI:** 10.7717/peerj.13416

**Published:** 2022-06-13

**Authors:** Zanri Strydom, Lauren J. Waller, Mark Brown, Hervé Fritz, Kevin Shaw, Jan A. Venter

**Affiliations:** 1 Department of Conservation Management, Faculty of Science, Nelson Mandela University, George, South Africa; 2 REHABS International Research Laboratory, CNRS-UCBL-NMU, Nelson Mandela University, George, South Africa; 3 Department of Biodiversity and Conservation Biology, University of the Western Cape, Bellville, South Africa; 4 Southern African Foundation for the Conservation of Coastal Birds (SANCCOB), Cape Town, South Africa; 5 School of Life Sciences, University of KwaZulu-Natal, Durban, South Africa; 6 Scientific Services, CapeNature, Cape Town, South Africa

**Keywords:** Selective culling, Endangered seabird, Seal-seabird predation, Fish biomass, Conservation concern, Fledgling mortality, Predation probability, Predator control, Hydroacoustic survey

## Abstract

Seabird populations experience predation that can impact their breeding density and breeding success. The Cape gannet *Morus capensis* is endemic to the Benguela upwelling ecosystem and is classified as Endangered by the IUCN. They are affected by several threats, including predation by the Cape fur seal *Arctocephalus pusillus pusillus*. Many fledglings succumb to predation during their maiden flight across waters around the island. To curb predation, the selective culling of individual predatory seals was implemented in 2014, 2015, and 2018. Our first study objective was to determine if selective culling of Cape fur seals significantly reduced predation probability on Cape gannets. We tested whether predation probability in 2014, 2015, and 2018 was affected by fish biomass, gannet fledgling numbers, and/or the presence/absence of selective culling. Our second objective was to determine what led to fluctuations in Cape fur seal predation on Cape gannet fledglings between 2007 and 2018. We tested whether fish biomass and the amount of Cape gannet fledglings in the water affected predation probability on the fledglings. Results indicated that selective culling reduced predation within years. We found that with both increased fledgling numbers and increased fish biomass, seal predation probability was reduced. This suggests that a sustainable way to promote the conservation of Cape gannets would be to increase food availability for both the Cape fur seals and Cape gannets. Our findings, collectively with the global trend of the declining Cape gannet population and their endemism, provide reasons advocating for the conservation of the food resources of both the Cape fur seal and the Cape gannet in the Benguela system.

## Introduction

Pinniped predation on seabirds was initially thought to occur only as an act of opportunistic hunting (David et al., 2003), however recently the extent of predation has created conservation challenges (Swan et al., 2020). Since 1990’s there has been a large increase in predation of Cape fur seals Arctocephalus pusillus pusillus on seabirds (Makhado, Crawford & Underhill, 2006). This increase is coincidental with both an increase in the seal populations (Makhado, Crawford & Underhill, 2006) and mismatched distributions of their pelagic prey species anchovy Engraulis capensis and sardine Sardinops sagax (Navarro, 2000). Although only a small proportion of seal individuals actively prey on seabirds, the impact thereof can rapidly threaten a seabird population (Swan et al., 2020) through desertion of breeding attempts (Wolfaardt & Williams, 2006), decreased survival (Horswill et al., 2016) and a decrease in recruitment (Sherley et al., 2019). In southern Africa, predation by Cape fur seals on seabirds has been recorded around six islands, with an additional five locations in Namibia (David et al., 2003; Kirkman, 2009; Makhado et al., 2009). In the Benguela ecosystem off the West Coast, Cape fur seals are known to prey on at least five seabird species (David et al., 2003), four of which are endangered. In a single breeding season, seals were responsible for 10,800 successful predation events on seabirds at Malgas Island (110 km from Lambert’s Bay on South Africa’s West Coast; home to 31,000 breeding pairs at the time; Crawford et al., 2007; Makhado, Crawford & Underhill, 2006). This research revealed that Cape fur seal predation on seabirds can be extensive and is, consequently, a cause for conservation concern (Makhado, Crawford & Underhill, 2006).

The Cape fur seals have a global population size of 1,060,000 individuals and they are globally classified as Least Concern by IUCN (Hofmeyr, 2015). Their population benefits from both legal protection against seal harvesting (David et al., 2003) and the fishery waste discarded in South African waters (Wickens et al., 1992). The Cape fur seals’ population resilience is aided by their generalist foraging behaviour, feeding on a wide variety of fish, prawns, lobster, shrimp (David, 1987), squid (Connan et al., 2014), and seabirds (Kirkman, 2009; Sherley et al., 2019) including the Cape gannet *Morus capensis*.

Predation on the Cape gannet is of great concern. With a current global population of 246,000 individuals, having undergone a 51.5% population decline between 1956 and 2015, they are classified as Endangered (BirdLife International, 2018). Additional threats to their population include, but are not limited to, an increase in pollution caused by industrial effluents and oil spills (Du Toit & Bartlett, 2001), and a reduction in food resources from overfishing and prey distributional shifts (Crawford et al., 2014; Blamey et al., 2015). Anchovies and sardines are the main prey species of the Cape gannet (Pichegru et al., 2009). Factors that influence the distribution and abundance of sardines and anchovies include climate fluctuations, which cause large-scale changes in ocean temperature (Chavez et al., 2003) and fishing pressure (Blamey et al., 2015). Since the early 20th century, the bulk of the sardine biomass shifted in distribution from the west of Cape Point to the east thereof (Coetzee et al., 2006; Coetzee et al., 2008b). These changes can result in poor breeding success of the West Coast Cape gannets as the prey species’ abundance decline or their distribution shifts beyond the foraging range of the birds’ (*e.g.*, Crawford et al., 2008; Kirkman, 2009). As the sardine and anchovy are also an important food source for Cape fur seals (David, 1987), depleted fish populations may increase predation risk of Cape gannets by Cape fur seals as the seals switch to alternative prey species such as seabirds (Marks, Brooke & Gildenhuys, 1997). This prompts the question; are seabird populations affected by being predated?

Selective culling was executed at Malgas Island to control seals found attacking seabirds at their breeding sites and preying on Cape gannet fledglings (David et al., 2003; Kirkman, 2009; Makhado et al., 2009). Predation on seabirds can be driven by prey fish availability but this type of predation has also resembled a play behaviour (Marks, Brooke & Gildenhuys, 1997) whereby opportunistic seals play with the seabird and kill it in the process. Seal predation on fledglings is a cause for concern as the Cape gannet survival may be influencing population trends at some colonies (Distiller et al., 2012). The implementation of culling has provided evidence of its effectiveness in reducing Cape gannet fledgling mortalities within a breeding season (David et al., 2003; Makhado et al., 2009) but we are uncertain about the effectiveness of culling between seasons (from one breeding season to another). Some evidence suggests that where bird predation is limited to a small number of animals, their prompt removal can entirely remove the problem (Moore, Charteris & Larsen, 2008). However, where the behaviour is established especially in a pinniped population, selective culling may reduce bird mortality rates (David et al., 2003; Du Toit et al., 2004; Makhado et al., 2009; Weller et al., 2016). If predation is not managed, it can be consequential.

To mitigate predation of Cape gannets at sea by Cape fur seals at Lambert’s Bay, the factors driving predation need to be understood, and management interventions must be assessed. Here we determined, firstly, whether selective culling of Cape fur seals significantly reduced predation probability on Cape gannets, and secondly, determine factors influencing fluctuations in predation rate by Cape fur seals on Cape gannet fledglings between 2007 and 2018.

## Methods

### Study area

**Figure 1:**
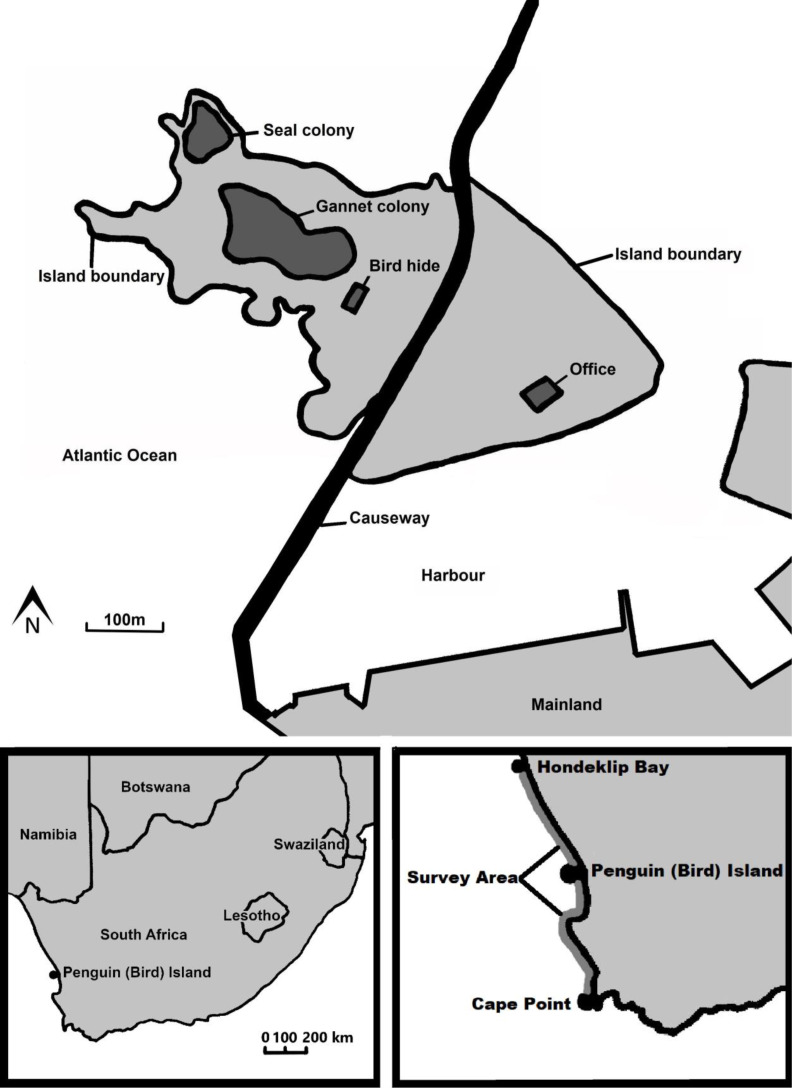
A map of the features on Penguin (Bird) Island including both a map of where the island is situated in southern Africa and where the fish biomass survey areas are.

Penguin (Bird) Island (32°5′24.43″S, 18°18′9.47″E) is located in Lambert’s Bay, Western Cape Province, South Africa. It is situated on the Atlantic coast in the Benguela upwelling system (Koné et al., 2005). The island, a provincial nature reserve manged by CapeNature (2016), measures three hectares (Duffy & La Cock, 1985) and is connected to the mainland *via* a man-made causeway (Jarvis & Cram, 1971) (Fig. 1).

### Culling

During 2014, 2015, and 2018, selective culling was implemented by the local provincial conservation authority on Cape fur seal individuals who were observed preying on Cape gannet fledglings in the water (Table 1). Culling was implemented only in these years due to logistical difficulties. Some seals specialise in predation on seabirds which is a learned behaviour where the young are taught by older individuals (Du Toit et al., 2004; Kirkman, 2009; Marks, Brooke & Gildenhuys, 1997; Makhado et al., 2009). Predation on seabirds had to be observed before the responsible seal could be culled, to ensure that culling was selective. The non-culling period is defined as the time from the first day that fledgling predation was observed to the day before the first day that culling occurred in that specific breeding season, noting that culling did not occur every day once culling started in a season. The culling period extends from the first day that culling occurred to the last day of the fledging period in that specific breeding season. The time and effort spent waiting for predations (before being allowed to selectively cull) varied daily within each year. The annual culling effort differed due to logistical difficulties and the availability of the CapeNature staff member that was licenced and responsible for the culling. Culling was approved and undertaken by CapeNature. Culling methods varied between years due to factors such as the weather and the availability of a boat. Culling took place either from a rubber duck (with a shotgun from a 10 m distance) or from land (with a triple two from an estimated 100–200 m distance) which ensured a humane death. At most, 0.7% of the annual adult Cape fur seals from the seal colony were culled and therefore not likely to have impacted their population trajectory. Culling was implemented once the necessary licenses and permits had been obtained.

**Table 1:** Information on culling periods in 2014, 2015 and 2018. We include the percentage of Cape gannets that fledged in the culling and non-culling period, the date of implementation and how many Cape fur seals were shot at Lambert’s Bay Cape gannet colony, South Africa.

Culling year	Number of fledging days	Fledging days part of culling period	% fledged in culling period	% predated in culling period	Date(s) of culling	Average daily adult seals in seal colony	Number of seals shot
2014	105	37	78% (*n* = 3999)	21% (*n* = 183)	13 and 14 April	1517 (*n* = 35)	8
2015	117	84	42% (*n* = 3164)	18% (*n* = 80)	13 and 19 March	2135 (*n* = 68)	15
2018	119	64	94% (*n* = 6502)	2% (*n* = 4)	19 April	1502 (*n* = 52)	2

### Seal and gannet observations

The number of Cape fur seals on the island at the seal colony was recorded by CapeNature staff (the observers) every morning before 08:00. Their population size was estimated from a high vantage point near the colony. The observers counted the number of juvenile Cape gannets that had fledged from the colony into the ocean, and predation events were recorded daily from 07:00 to 19:00 between 2007 and 2018. The observers rotated shifts hourly to avoid fatigue and lapses in concentration. The water surrounding the island was scanned for predation activity using both the naked eye and binoculars. To prevent encroachment of Cape fur seals into the gannet colony, the observers actively restricted the seals’ access by displacing invading seals from areas adjacent to the Cape gannet colony by vocal noises or hand clapping. This forced the seals to return to their own colony on the island or to the ocean.

**Figure 2:**
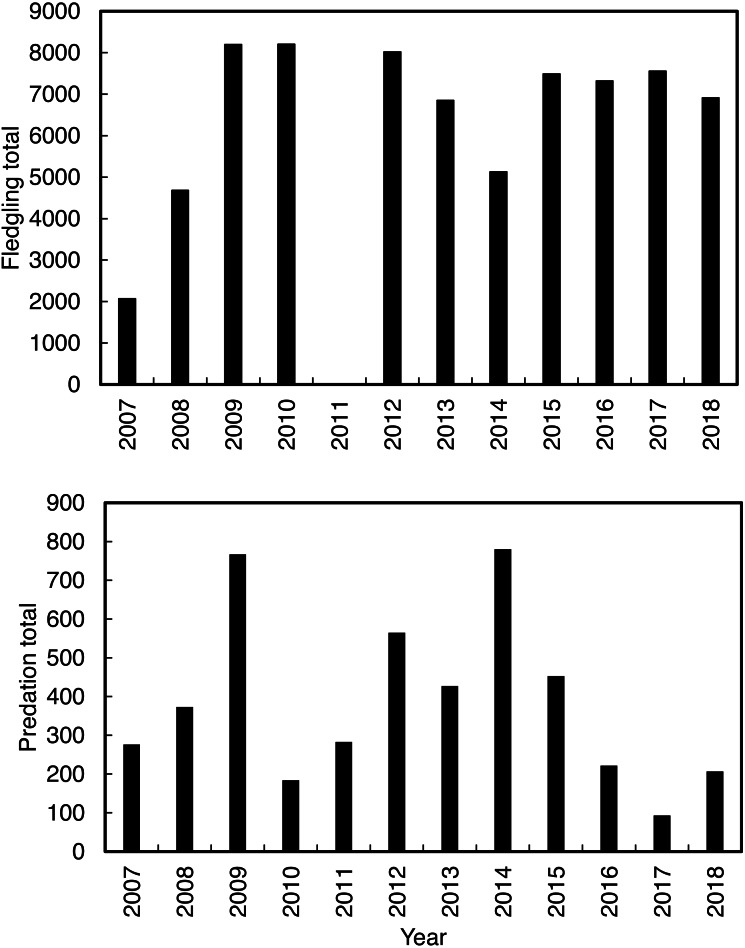
The total annual Cape gannets that fledged successfully (excluding 2011; without being predated) and the annual total predations by Cape fur seals on Cape gannet fledglings from 2007 to 2018 at Lambert’s Bay gannet colony, South Africa.

Annual predation of Cape gannet fledglings (Fig. 2) and fledgling abundance were recorded by the observers from January to June between 2007 and 2018. Fledgling numbers were not recorded in 2011 due to low staff availability. Predation probability was measured by calculating the number of fledglings observed being preyed on, in proportion to the total number of fledglings from 2007 to 2018. Predation usually occurs when a fledging Cape gannet, on its maiden flight from the island, lands on the water near the island. Some fledgling gannets are successful in their maiden flight (meaning that they fledged off the island without being predated), while the unsuccessful individuals return to the island for another attempt. To prevent double counting, young gannets were only considered as successfully fledged once they flown/swum more than 300 m from the shore. The work obtained ethics approval (ethics clearance reference number A18-SCI-SNRM-003) issued by the Nelson Mandela University. The work was conducted under a research permit (permit number CN32-30-4824) issued by CapeNature.

### Fish biomass availability

Fish biomass data were obtained to determine the link between the amount of main food species available and the amount of fledglings predated by Cape fur seals. The summer (October to December) hydro-acoustic survey biomass estimates of anchovy and sardine were obtained from the Department of Forestry, Fisheries and Environment. These surveys estimate the biomass of the adult fish between Hondeklip Bay on the West Coast and Port Alfred on the East Coast of South Africa (Barange, Hampton & Roel, 1999; Coetzee et al., 2008a). For the purpose of this study, we used the biomass for the stratum overlapping the foraging range of local Cape gannets (Grémillet et al., 2004). This overlap covers the area between Hondeklip Bay (290 km north of Lambert’s Bay) and Cape Point (310 km south of Lambert’s Bay) (Fig. 1; Moseley et al., 2012). Surveys were conducted according to a pre-stratified random survey design. This design aims to reduce sampling variance by grouping areas of known similar density into strata. Within each stratum, a predetermined number of randomly-spaced parallel transects, perpendicular to the coastline, are surveyed (Barange, Hampton & Roel, 1999). These surveys, which use sphere-calibrated Simrad scientific echo-sounders installed on the research vessels, have been conducted annually since 1984 (Hampton, 1992). Acoustic estimates of mean fish density are derived for each transect from measures of acoustic intensity according to methods described by Coetzee et al. (2008a). Fish biomass for each stratum was estimated from the mean density of fish encountered in each transect, and weighted by the transect length (Hampton, 1992).

### Statistical analysis

Our data were analysed to determine the influences (if any) of culling and fish biomass on Cape gannet fledgling predation probability (the response variable). In our generalised linear model (GLM), the presence/absence of culling, annual total fish biomass, and the annual total number of fledglings were set as the explanatory variables. To standardize the variables, we used the beta(model) function in the Reghelper package (Hughes, Beiner & Team, 2017) in R version 3.5.3 (R Core Team, 2017). The total fish biomass was the sum of the sardine and anchovy biomass between Hondeklip Bay and Cape Point. The annual total number of fledglings was determined by the sum of the daily successful fledglings counted within a year (this excludes the number of predations). Culling years were compared to years in which culling was not implemented. In our model, the reference level was set to the years during which culling was not implemented. With data from 2007 to 2018 (excluding 2009 and 2011, *N* = 10 years), the response variable was set to the annual predation probability. The fish biomass datapoint reported for 2009 was an outlier based on Cook’s distance, even after standardising, and therefore it was excluded from the GLM (see Supporting Information Table S1). Weather conditions were excluded from analysis as the desired small-scale site specific data are not available. Previously it has been suggested that wind affect predation on gannet fledglings. It is thought that fledglings mainly fly off during windy conditions rather than jump into the water, as they do on calm day where they then get predated on Makhado, Crawford & Underhill (2006).

Since the results of only three culling years are inconclusive, we have forecasted the gannet population with and without culling for twenty years using variations within our own original dataset. The sole objective of the population forecast is to compare the population size during years in which no culling was implemented (Table S3), compared to the consistent implementation thereof (Table S4). Our equation included annual gannet population numbers (from 15,000 individuals in year 1) summed with annual fledgling numbers, minus annual mortality of 70 and 8% for fledglings and adults respectively (Wanless et al., 2006). Our forecast assumes that all fledglings recruit at their natal colony. The mean gannet population between 2006 and 2018 (as estimated by CapeNature) was 15,000 individuals hence we used it as our first gannet population value to base the equations on.

We used the observed *versus* the predicted values of the logistic regression model to provide us with an approximate R^2^ value of our model. Within-year comparisons of predation probability before and after culling were assessed separately. A *χ*^2^ contingency table comparison (Giese, 1996; Griffiths, 2002; Laerd Statistics, 2016) was run to compare predation in the culling and non-culling periods during the three years in which culling was implemented.

## Results

The GLM indicated that a higher predation probability was experienced in culling years compared to the non-culling years (Table 2). Because culling starts later in the season, we checked for potential confounding effects relating to delayed timing. Culling did not occur during a time when fewer Cape gannets fledged or when fewer Cape fur seals were present at the seal breeding colony, therefore we believe no confounding occurred. During the culling periods across the three years, the proportion of the Cape gannets fledged were 78%, 42%, and 94%, respectively (Table 1). The population forecast indicated that the gannet population in 20 years’ time will be larger (as a result of decreased predation) when consistent culling has been implemented, compared to when it has not been implemented.

**Figure 3:**
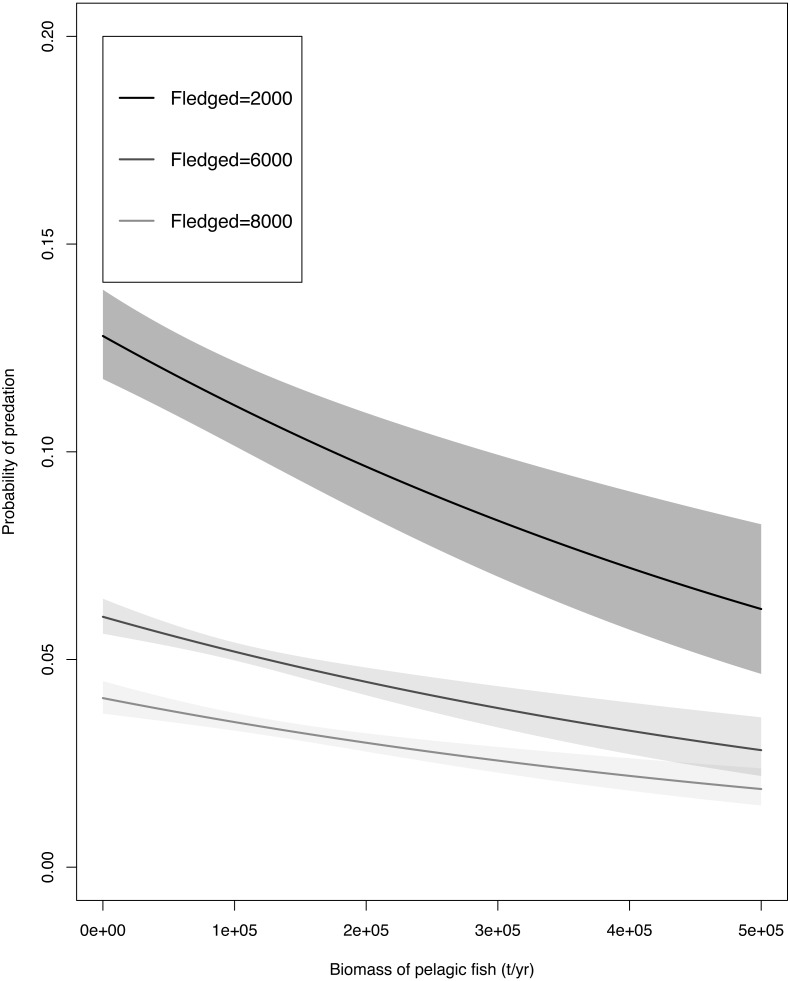
The generalised linear model predictions of the Cape gannet fledgling predation probability by Cape fur seals with a minimum of (*N* = 2,000), mean (*N*= 6,000) and maximum (*N* = 8,000) amount of Cape gannet fledglings with a change in fish bio.

Fish biomass and fledgling abundance had a weak correlation (*t* = 1.1017, *df* = 8, *p*-value = 0.3026, cor 0.3629527) hence both variables were used in our GLM. The GLM (Table 2) indicated that predation probability decreased with increased fish biomass and with increased total number of fledglings. The adjusted R^2^ value of the model was 0.568. The unstandardized data were used in Fig. 3 to portray a realistic trend, and the results table for this specific GLM can be found in the Table S2. The model predicts that under any given amount of fish biomass, the probability of a fledgling being predated was highest when the minimum number (*N* = 2000) of fledglings were available, intermediate when the mean number (*N* = 6000) of fledglings were available, and lowest when the greatest number of fledglings were available (*N* = 8000; Fig. 3).

**Figure 4:**
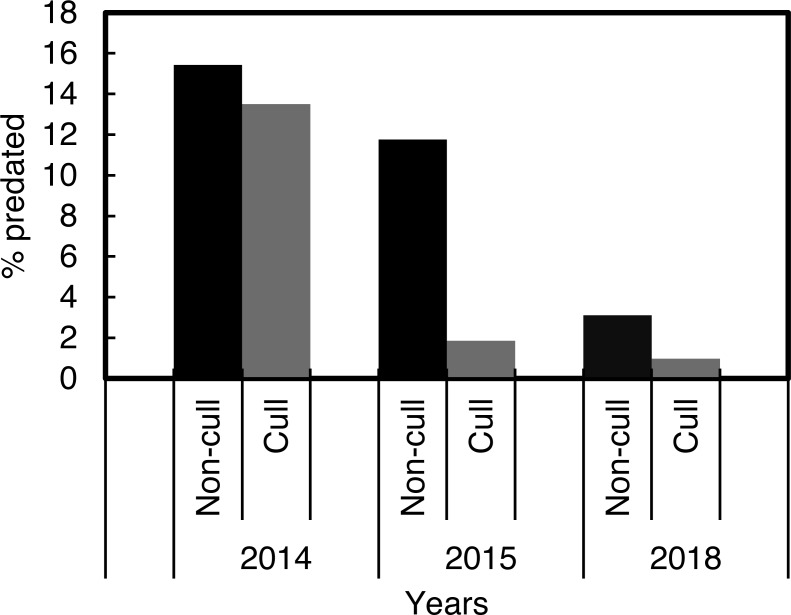
Percentage Cape gannet fledglings predated during the targeted Cape fur seal culling and non-culling periods in 2014, 2015 and 2018 at Lambert’s Bay gannet colony, South Africa.

The *χ*^2^ contingency table comparison, which was run to compare predation in the culling and non-culling periods during the three years, indicated that culling reduced predation rates within years, *χ*^2^ = 915.58, *df* = 10, *P* < 0.001 (Fig. 4).

**Table 2:** This presents the standardized results of the generalised linear model with a binomial distribution testing how the different factors influence Cape gannet fledgling predation probability between 2007 to 2018 (excluding 2009 and 2011). Predation probability is the response variable, and the explanatory variables are presence/absence of culling (its absence was the reference level), total fish biomass and the number of fledglings available in the water for the Cape fur seals to predate upon at Lambert’s Bay Cape gannet colony, South Africa.

Model	D.F.	Deviance	Resid. D.F.	Estimate	Standard error	*Z* value	*P* value
Intercept				−2.80546	0.01766	−158.840	0.001
Fish biomass	1	230.09	8	−0.11179	0.02239	−4.993	0.001
No. of fledglings	1	256.48	7	−0.38192	0.02181	−17.514	0.001
Culling	1	64.63	6	0.15650	0.01938	8.075	0.001

## Discussion

Seabird predation is a behaviour that only a few seal individuals show (David et al., 2003), yet even in their minority, they can be responsible for a large percentage of annual fledgling mortalities (Kirkman, 2009). We found that a higher predation probability was experienced in culling years compared to the non-culling years. However, the long-term effectiveness is tentative as it has not been implemented long enough to show that culling can significantly enhance or eliminate predation. We therefore forecasted the population of gannets both with and without culling for 20 years. The forecast indicated that the consistent culling will allow for a slightly greater population size than when culling is not being implemented. This is indicative that between years, if selective culling is implemented annually, it could lead to a larger population size than without it. In addition, we investigated the effect of culling within years, and found that the selective culling of seals reduced predation on Cape gannet fledglings. A reduction in predation will increase fledgling survival with a subsequent positive impact on the future population trajectory. In other studies, most seals that have predated on seabirds were younger than ten years of age (David et al., 2003; Makhado et al., 2009) hence it is hoped that persistent selective culling over five or more years may essentially eliminate predation on seabirds (David et al., 2003). To implement site-specific management measures over an effective timescale, the age of each predatory seal around the Cape gannet colony at Lambert’s Bay must be determined.

Despite Cape gannets being a long-lived seabird species (Distiller et al., 2012), the absence of predation pressure on juveniles is important in increasing survival rates in a population (Horswill et al., 2016), promoting population persistence. The prevention of a further decline in the gannet population will subsequently conserve genetic variation within the species, and it will maintain the effect of the dilution of predation risk (Roberts, 1996). Not only can predation directly be reduced but also indirectly through an increase in the prey populations (both the pelagic prey and Cape gannets). We found that in years with increased fish biomass, there was a subsequent decrease in predation probability. Worldwide, seabirds can be vulnerable to predators of different species, if their preferred natural (Marks, Brooke & Gildenhuys, 1997) or anthropogenic food source is reduced (Bicknell et al., 2013). However, research on Antarctic fur seals *Arctocephalus gazella* found that the seals frequently preyed on Macaroni penguins *Eudyptes chrysolophus* irrespective of the availability of their preferred prey species (Bonner & Hunter, 1982).

### Alternative predator control methods

We found that within the years during which culling was implemented, the culling of seals reduced the number of Cape gannet fledglings being predated. In addition to lethal methods, behavioural conditioning may be an alternate means to reducing predation. Electric pulse deterrents (Forrest et al., 2009), acoustic deterrents, and multi-transducer deterrents were found to effectively reduce the predation when implemented (Götz & Janik, 2013). Although seals can become habituated to particular deterring methods, continued explosions of firecrackers in the water were effective in deterring Cape fur seals from fishing nets (Shaughnessy et al., 1981). There are different methods of reducing predation on the Cape gannets, but the methods vary in terms of time, cost, and effectiveness.

### Fish biomass availability

Low prey biomass availability has been found to lower the growth rate of chicks, which influences survival probability (Grémillet et al., 2008). During years with low food availability, the juveniles’ condition in their maiden flight might be negatively affected, which could increase opportunities for predation. An increase in fish biomass may not only positively impact Cape gannet breeding success (Pichegru et al., 2009) and post-fledging juvenile survival but also decrease fledgling predation probability. This highlights the impact that increased food availability has on mitigating other threats to seabirds and the proportional impact that improving fish resources has on avoiding excessive predation on seabirds.

### Fish population management recommendations

An increase in food availability for the Cape gannets (and the Cape fur seals) could be the difference between a colony collapse and a growing population (*e.g.*, Hamer et al., 1993) as it serves, among others, to alleviate the predation pressure (*e.g.*, Heubeck, 2002). There are suggestions on the means with which to improve food availability for both Cape gannets and Cape fur seals. These include establishing Marine Protected Areas (MPAs) with dynamic boundaries (Hyrenbach, Forney & Dayton, 2000) around Cape gannet colonies and nearby breeding and foraging hotspots where these areas are closed to fishing (*e.g.*, Frederiksen et al., 2004; Pichegru et al., 2012). Dynamic boundaries would be essential to breeding seabirds as it will allow for ideal foraging conditions and the elimination of competition with commercial fisheries during the breeding season. Although we do acknowledge that having MPAs with dynamic boundaries could be difficult to enforce, as well as to monitor, and can have serious social implications (Sowman & Sunde, 2018). The effective management of MPAs provides a good tool that can be used to increase fish stock availability which yields good results for seabirds such as improved chick condition (Sherley et al., 2018) and a decrease in adult foraging effort (Pichegru et al., 2012). This indicates that a no-take zone can be essential to breeding birds that rely on pelagic prey (Pichegru et al., 2012). Alternate ways of improving food security for seabirds include implementing fish resource conservation measures such as: developing and enforcing realistic fishing catch quotas (Tasker et al., 2000) and incorporating an adequate ecological buffer in fishing quotas which makes provision for ecosystem requirements (Cury et al., 2011; Pichegru et al., 2012).

The continued over-exploitation of local forage fish populations is likely to increase seal predation probability on seabirds. Reductions in predation through selective culling are short-lived (Makhado et al., 2009; Bowen & Lidgard, 2013), and therefore, a more sustainable option would be to increase fish stock conservation efforts in this region. Not only will an increase in fish biomass result in an improvement in food availability for the Cape gannets but also for the Cape fur seals. Abundant mammals can affect threatened seabird species (David et al., 2003), therefore, sustainable management of food availability for both species should be a priority as seal predation on seabirds decreased with an increase in pelagic prey biomass. Until the fish resource availability has improved (at least more than double the current sardine trends; *e.g.*, between two and four million tonnes of biomass (Coetzee et al., 2008b)), and conservation thereof is effective, it might be advantageous for the Cape gannet population if predator management continues. Our findings, collectively with the global trend of the declining Cape gannet population and their endemism, provide reasons advocating for the conservation of the food resources of Cape fur seals and Cape gannets in the Benguela system.

##  Supplemental Information

10.7717/peerj.13416/supp-1 Supplemental Information 1 The standardized results of the generalised linear model with a binomial distribution testing how the different factors influence Cape gannet fledgling predation probability between 2007 to 2018 (including 2009)

10.7717/peerj.13416/supp-2 Supplemental Information 2 The results of the unstandardized generalised linear model with a binomial distribution testing how the different factors influence Cape gannet fledgling predation probability between 2007 to 2018 (excluding 2009 and 2011)

10.7717/peerj.13416/supp-3 Supplemental Information 3 Cape gannet population forecast for 20 years (with 15 000 individuals in year 1) in the absence of culling Cape fur seals while accounting for an annual 70% and 8% mortality of fledglings and adults respectively (after Wanless et al., 2006) at Lambert’s

10.7717/peerj.13416/supp-4 Supplemental Information 4 Cape gannet population forecast for 20 years (with 15 000 individuals in year 1) while implementing culling of Cape fur seal while accounting for an annual 70% and 8% mortality of fledglings and adults respectively (after Wanless et al., 2006) at Lambert’s

## Additional Information and Declarations

### Field Study Permissions

The following information was supplied relating to field study approvals (i.e., approving body and any reference numbers): The work was conducted under a research permit (permit number CN32-30-4824) issued by CapeNature.

### Data Availability

The following information was supplied regarding data availability: The data that support the findings of this study are available from CapeNature and Department of Environment, Forestry and Fisheries (DEFF) but restrictions apply to the availability of these data, which were used under license for the current study, and so are not publicly available. Anchovy and sardine November biomass data from 2007 to 2018 were obtained from DEFF. The data were granted permission to Zanri Schoeman (maiden name) with the reference number: DATA2018/03. The data were made available by Dr Kim Prochazka (kimP@daff.gov.za; 0214023546). Data on Cape fur seal predation on Cape gannet fledglings and Cape fur seal culling data from 2007 to 2018 were obtained from CapeNature. The data were granted permission to Zanri Schoeman (maiden name) but no reference number was associated with the application. The data were made available by Therese Forsyth (tforsyth@capenature.co.za).
