# Supplementary material for: Factors that influence Cape fur seal predation on Cape gannets at Lambert’s Bay, South Africa
Source: PeerJ. 2022 Jun 13;10:e13416. doi: 10.7717/peerj.13416 (PMC9202551; doi:10.7717/peerj.13416)
Supplement: Supplemental Information 1 [file peerj-10-13416-s001.docx]

Table S1: The standardized results of the Generalised Linear Model with a binomial distribution testing how the different factors influence Cape Gannet fledgling predation probability between 2007 to 2018 (including 2009). Predation probability is the response variable, and the explanatory variables are presence and absence of culling, total fish biomass and the number of fledglings available in the water for the seals to predate upon at Lambert’s Bay Cape Gannet colony, South Africa.

| **Generalised Linear Model** | **Degrees of Freedom** | **Estimate** | **Standard error** | **Z value** | **P value** |
| --- | --- | --- | --- | --- | --- |
| Intercept |  | -2.74673 | 0.01616 | -169.98 | 0.001 |
| Number of fledglings | 8 | -0.39882 | 0.02201 | -18.12 | 0.001 |
| Culling | 7 | 0.24055 | 0.01888 | 12.74 | 0.001 |
| Fish biomass | 6 | 0.28120 | 0.02336 | 12.04 | 0.001 |
