# Supplementary material for: Factors that influence Cape fur seal predation on Cape gannets at Lambert’s Bay, South Africa
Source: PeerJ. 2022 Jun 13;10:e13416. doi: 10.7717/peerj.13416 (PMC9202551; doi:10.7717/peerj.13416)
Supplement: Supplemental Information 3 [file peerj-10-13416-s003.pdf]

Table S3: Cape gannet population forecast for 20 years (with 15 000 individuals in year 1) in the absence of culling Cape fur seals while accounting for an annual 70% and 8% mortality of fledglings and adults respectively (after Wanless et al. 2006) at Lambert's Bay gannet colony, South Africa.

Year	Fledgling number	Fledgling mortality	Adult mortality	Gannet population
1	7555	5289	1200	16066
2	5080	3556	1285	16305
3	7322	5125	1304	17197
4	2066	1446	1376	16442
5	4679	3275	1315	16530
6	6854	4798	1322	17264
7	8020	5614	1381	18289
8	8203	5742	1463	19287
9	6222	4355	1543	19611
10	8203	5742	1569	20503
11	2066	1446	1640	19483
12	2066	1446	1559	18544
13	6538	4577	1484	19021
14	5760	4032	1522	19228
15	2066	1446	1538	18310
16	8020	5614	1465	19251
17	2066	1446	1540	18331
18	6222	4355	1466	18731
19	5500	3850	1499	18883
20	8203	5742	1511	19833
