# Supplementary material for: Factors that influence Cape fur seal predation on Cape gannets at Lambert’s Bay, South Africa
Source: PeerJ. 2022 Jun 13;10:e13416. doi: 10.7717/peerj.13416 (PMC9202551; doi:10.7717/peerj.13416)
Supplement: Supplemental Information 4 [file peerj-10-13416-s004.pdf]

Table S4: Cape gannet population forecast for 20 years (with 15 000 individuals in year 1) while implementing culling of Cape fur seal while accounting for an annual 70% and 8% mortality of fledglings and adults respectively (after Wanless et al. 2006) at Lambert’s Bay gannet colony, South Africa.

Year	Fledgling number	Fledgling mortality	Adult mortality	Gannet population
1	7555	1446	1200	14420
2	5080	3275	1130	14694
3	7322	3556	1112	15106
4	2066	5614	1172	16340
5	4679	4798	1250	17146
6	6854	5125	1278	18065
7	8020	5289	1267	19063
8	8203	4157	1282	19563
9	6222	1446	1248	18936
10	8203	5614	1124	20218
11	2066	4478	1192	20945
12	2066	1446	1268	20298
13	6538	1446	1132	19785
14	5760	5614	1049	21142
15	2066	3807	1192	21582
16	8020	4157	1245	22119
17	2066	1446	1207	21532
18	6222	1446	1124	21029
19	5500	5614	1049	22385
20	8203	3672	1192	22767
